# WhatsApp Messenger use in oncology: a narrative review on pros and contras of a flexible and practical, non-specific communication tool

**DOI:** 10.3332/ecancer.2021.1334

**Published:** 2021-12-13

**Authors:** Vittorio Gebbia, Dario Piazza, Maria Rosaria Valerio, Alberto Firenze

**Affiliations:** 1 Medical Oncology Unit, La Maddalena Clinic for Cancer, Palermo, Italy; 2 Department of Internal Medicine ‘Promise’, University of Palermo, Palermo, Italy; 3 GSTU Foundation for Cancer Research, Palermo, Italy; 4 Medical Oncology Unit, Policlinic ‘P. Giaccone’, University of Palermo, Palermo, Italy; 5 Risk Management Unit, Policlinic ‘P. Giaccone’, University of Palermo, Palermo, Italy

**Keywords:** WhatsApp, instant messenger systems, social media, telemedicine, cancer care, health professional’s interaction, cancer research

## Abstract

The spread of instant messenger systems provides an excellent opportunity and a helpful tool to healthcare professionals. WhatsApp instant messenger use is widely prevalent among health professionals, cancer patients, caregivers and the general population. It is a quick and easy communication tool that may also be used on personal computers and business purposes. WhatsApp instant messenger and other similar tools may be a very useful complement for e-medicine. Instant messaging systems may be helpful, especially in rural areas, in medium- or low-income countries, or to avoid unnecessary travels, improve knowledge and awareness of cancer, monitor home care and support the delivery of home care. The unregulated use of WhatsApp instant messenger requires sound and shared guidelines to assure impeccable professional service. Although a significant number of papers have investigated the roles of social networks in connecting patients to health professionals, there is still a lack of information and scientific data about their uses, benefits and limitations in connecting health providers only for professional communication. The role of instant messenger systems in cancer practice and research needs to be clarified. In this paper, we report a focus on available data, pros and contras of the unregulated use of WhatsApp instant messaging, in the context of e-medicine, as an interprofessional and doctor/patient communication tool in oncology.

## Introduction

In the last decade, web-based technology has conquered an ever more significant space in oncology healthcare organisations and scientific research worldwide [1]. The COVID-19 pandemic has caused a profound alteration or even disrupted the usual ways of communication between patients, caregivers, health professionals and oncological institutions [2]. In this tragic scenario, telemedicine and web-based tools have undergone an even more notable expansion than usual. These tools can minimise face-to-face contacts, avoid unnecessary travels and provide fast and efficient interprofessional and patient/doctor communication [3–6]. Epidemiological data on the rise of cancer incidence and prevalence further enhanced this trend. The American Society of Clinical Oncology (ASCO) predicted the number of cancer survivors would rise from 15.5 million to 20.3 million between 2016 and 2026 [7]. The growing prevalence of cancer survivors combined with the rise in cancer incidence will raise a nearly 50% increase in cancer care demand which can be challenging to manage for the upcoming possible shortage of oncologists, at least in industrialised countries [8]. Hopefully, telemedicine can help healthcare systems cope with the overwhelming burden of patients numbers [9].

The spread of social media and instant messenger systems provides an excellent opportunity and a helpful tool to healthcare professionals [10]. The Merriam-Webster dictionary defines social media as ‘forms of electronic communication through which users create online communities to share information, ideas, personal messages, and other content’ [11]. This definition may also apply to instant messenger systems, which are not the same as social media, but they are closely interrelated and somehow fade into each other. Among these tools, WhatsApp instant messaging (WM) system gained a vast diffusion worldwide. WM is a free messaging application, multiplatform, which allows users to send textual messages, images, videos, documents and voice recordings to other users via the Internet, having the telephone number as an identifier [12]. It also allows users to make voice and video calls, send images and documents and is compatible with all smartphones. It differs from ‘chat groups’, in which the user engages in a more public real-time conversation within a chatroom where everyone on the channel sees everything said by all other users.

The use of a fast-acting, written or video communication system with an app already well known, like WM, is conveniently within reach of a large majority of patients due to its widespread use in peoples’ private daily lives. Due to its wide diffusion, there is no need to ask the patient or the caregiver to download or create an account on other Apps, which could be a barrier. These factors have caused a massive boost in web-based educational meetings and the increasing use of telematics communication methods among patients, families, hospitals and different health professionals of the same speciality or belonging to multi-disciplinary teams [10, 13, 14]. However, today an ever-increasing number of apps are offered to oncological health professionals by various providers to provide an efficient patient problem management system [14, 15]. Scottish researchers reported an analysis of 151 apps targeting people living with and beyond cancer covering various aspects of oncology ranging from disease information and planning medical care to self-monitoring, management strategies and interaction between users and professionals [3, 15]. Nearly two-thirds of tools have been developed by commercially oriented companies/private individuals, and one-third by non-profit associations, mainly in the USA. Some of the apps are ethically and professionally correct and useful in managing specific clinical settings such as oral cancer therapies, palliative care, exercise and many others [16]. Unfortunately, many apps exploit an engagement system based on the veiled promise, sometimes exaggerated, of empowerment in the struggle against the disease. Furthermore, a minority of apps deplorably report unproven ‘cures’ for cancer or selling products, such as alkaline waters, to cancer survivors.

In the traditional face-to-face medical visit, the empathic translation of clinical evaluation flowcharts and medical concepts into simple, friendly and non-technical language easily understood by patients and their caregivers represents a cornerstone of the medical art. However, e-medicine communication/information systems often follow a medical logic workflow that is often difficult to interpret by many potential non-medical users and patients for various intuitive reasons. However, the ambitious goal of optimising communication in health care is challenged by the conceptual complexity of many apps, mainly unknown to the general population. For this reason, very often, oncological health professionals have turned to the use of web-based communication and messaging systems already widespread in the world and well known to most of the possible users. Among these tools, the WM instant messenger system has gained enormous popularity due to ease of use, versatility and almost additional costs.

In the last years, the research community analysed the advantages and disadvantages of WM usage in healthcare. Among the negative aspects, scientists included the clinical risks for patients, data security and privacy protection. A working group of the ASCO, the Integrated Media and Technology Committee, reported some suggestions on the responsibly professional use of social media in oncology [17]. While lacking defined guidelines, however, the committee discussed some critical key-issues, including respect for patients’ privacy, protection of sensible clinical data in compliance with the legal, regulatory rules and the possible institutional property of communication tools [17].

In this narrative review, we report a focus on available data, pros and contras of the unregulated use of WM, in the context of e-medicine, as an interprofessional and doctor/patient communication tool in oncology.

### WhatsApp Messenger and business application programming interface (API)

WM allows health professionals to share data, updates, information, debate health care policy and practice issues [14]. WM also may be used to promote health behaviours, engage with the public and educate and interact with patients, caregivers and students, and colleagues. Although some messaging systems are very similar, if not identical, social media, however potentially carry the additional advantage of being a very efficient management system, is still scientifically unproven in oncology [14, 17]. WM may also be used from any computer station in the WhatsApp Web version that recognises a quick response QR code and connects directly to the user’s smartphone. As inferred from the lack of medical literature, not many oncologists know the business version of WM, a free version for smartphones valid for professionals, which permits communication with all standard version WM users [18]. The main difference between regular WM and the Business application is in the profile window. While the standard version contains only name, status and photo, the dedicated business app shows an actual business card, with all the helpful information for being recognised and found by customer such as name, logo, image, description, opening and closing times, website, e-mail address, etc. WM Business API allows appointment management, request medical or laboratory reports, symptoms evaluation, answers to FAQs, statistical analysis, automatic responses and guided workflows, organisation of contacts by categories and broadcast list. In the latter action, the app guarantees individual users’ privacy since individual responses are blinded to other users. Healthcare organisations can store massive medical data of patients by integrating WM Business API with other third-party tools.

### Health professionals-related issues

A review by Brazilian investigators reported that WM is a helpful communication tool between health care professionals and patients, caregivers and families, among health care professionals themselves, or as a learning tool for providing information on health care to professionals or the general population [19]. However, the authors felt that high-quality and adequately evaluated research is urgently needed. Patt [5] explored the use of telemedicine and barriers during the COVID-19 pandemic in a statewide, multicentre, high patient volume oncology network. Overall, 96% of clinicians reported telemedicine, with 33% using it for more than 25% of patient visits. Most clinicians said that patients enjoy the benefits of telemedicine, primarily those minimally symptomatic. Its use may cause a decrease in exposure risk and transportation needs. Moreover, it enhanced the presence of caregivers during patients’ visits. Availability of technical equipment and Internet resources, such as broadband access, was the most frequent obstacle to patients’ access to telemedicine.

#### Privacy

WM has an encryption system for both written and video messages to guarantee privacy [13, 18]. Despite this protection system, several researchers formulated warnings and concerns on the unregulated use of instant messenger systems in health care [1, 19, 20]. Health authorities expressed significant concern concerning confidentiality in WM conversations to avoid putting patients in a vulnerable position and maintain the public’s trust in the medical profession [20]. However, the reality is that many doctors regularly use instant messaging systems to communicate data and information and prescriptions to patients even in the face of regulatory agencies’ recommendations. A recent Irish study reported that 97% of doctors surveyed shared sensitive information on instant messenger without the patient’s written consent, although 68% were concerned about this lack of agreement [20]. The use of WM can also carry the risk for health professionals of becoming the target of offenses that may spread on the web [21].

#### Video-consulting

The use of WM for patient engagement has begun to appear more and more in medical literature [22]. From a technical point of view, a video consultation is a simple video call. Therefore, different platforms can also be used, including well-known and widespread systems (WhatsApp, Google Hangout, Zoom and many others), or customised and private solutions. For a professional who starts the video consultation activity independently, the best advice is to use the business version WM, which represents a professionally-oriented tool [18].

#### Interprofessional cooperation

WM may be particularly helpful for interprofessional cooperation. The Italian College of Medical Oncology Directors carried out a descriptive, unplanned investigation to report reactions, attitudes and countermeasures put in place and implemented by 19 medical oncology units facing the COVID-19 outbreak in Southern Italy [23]. All the directors of medical oncology units participate in an informal WM chat. Authors reported 956 WM conversations related to reactions to important events, such as the epidemic spread, Government ordinances and guidelines during 4 weeks of observation. Data showed significant awareness of problems linked to the COVID-19 epidemic among medical oncologists and rapid diffusion of countermeasures. Actions taken were correlated time-wise to important events. Data showed that the number of WM conversations correlated with the volume of activity of oncology units. Textual sentiment analysis of raw data showed a reduction of positive emotions over the weeks. A significant increase in negative emotions was observed as the outbreak impacted the healthcare system. Shaarani *et al* [24] investigated the prevalence of WM use as an interprofessional communication tool among 5,329 Lebanese physicians explored the dimensions of its use through an e-mail-based questionnaire. Overall, 429 physicians responded completed the survey and 96.5% of them reported using WM, 72.7% had WM consultations with colleagues and about 50% said being members of professional WM groups. Although most participants state to avoid patient identification when sharing information, two-thirds of physicians felt it necessary to develop guidelines, especially for the medico-legal and ethical aspects. The creation of social media-based groups may enhance interprofessional networking and sharing of knowledge among different specialities. Rolls *et al* [25] published a review of 44 studies present in the medical literature. Overall, the quality of studies was not optimal, and data collection included web-based observation, surveys, interviews, focus groups and diaries. Health care professionals involved in the studies included physicians, nurses, allied health professionals, followed by health care professionals in general, a multi-disciplinary clinical speciality area and midwives. Analysis based on related theories of ‘planned behavior’ and the ‘technology acceptance model’ suggests that social media use is dependent on an individual’s positive attitude toward and accessibility of the media, which credible peers reinforce. The most frequent driver to create a virtual community is the need for an agorà where health professionals and other users may share and discuss relevant speciality knowledge to enable themselves to make updated practical decisions. Chat participants showed a frequent attitude to read or access speciality-specific clinical information but a low tendency to post data or opinions. Hospitals can leverage the considerable potential of WM to connect with their patients and to organise efficient team coordination. For instance, WM may answer all the necessary information or status of each piece of equipment or coordinate actions. Italian investigators carried out a study aimed to assess the individual and organisational determinants that can trigger or inhibit the use of WM in a hospital setting and which variables managers can exploit to guide professionals’ behaviours [26]. A validated questionnaire tested for internal consistency was administered to 191 physicians and nurses in an academic hospital showing that WM is widely used in the hospital due to the subjective perception of its usefulness and numerous practical advantages. Data show that WM may decrease consultation time, number and time spent on phone calls, improve communication and rationalise workflows. The creation of WM groups promotes collaboration, ultimately improving the level of healthcare provided to patients. Moreover, an interplay exists between organisational and individual factors in determining the use of WhatsApp between healthcare professionals and patients. In particular, individual factors play a crucial role as determinants of WM. In contrast, organisational factors play a secondary role, not directly influencing the use of WM but always acting through individual elements. This study also analysed the influence of personal and organisational determinants of WM use in the hospital setting. It provides hospital managers with vital information to manage this phenomenon and implement adequate strategies to increase its potential. An extensive literature review aimed to examine the utilisation of social networks for health professionals’ relationships, daily clinical practice, professional networks and education and training to identify areas for future health communication research [13]. Overall, 33 out of 6,977 papers retrieved stated that social networking systems enhanced effective communication and information sharing even if sound scientific evidence lacks. Health professionals used social networking systems to support delivering clinical services, make referrals and share information. They were beneficial to network building and professional collaboration. Social media were considered novel tools to enhance educational interactions among peers, students, instructors and preceptors. The application of social media came with restraints in technical knowledge, concerns on data protection, privacy and liability, issues in professionalism and data protection. Limitations included technical knowledge, professionalism and risks of data protection. The evolving use of such tools necessitates robust research to explore the full potential and the relative effectiveness in professional communication.

#### Burnout

Burnout syndrome and dissatisfaction affect more than 50% of practicing physicians and oncologists in the USA [27–29]. Only one paper suggests a possible benefit and may reduce the rate of physician burnout even if its use may be linked to pathological addiction [30]. Further studies are needed to clarify a role for instant messengers system in ameliorating burnout.

#### Research

Health providers and researchers can use data recorded in WM to find the best treatment options for patients, cooperate with clinical trials or verify patients’ journeys [1, 10, 14, 19]. The use of symptom checkers or chatbots to collect and analyse data directly from patients’ data on symptoms, family history and risk factors must be scientifically validated.

#### Teaching

Clavier *et al* [31] reported a prospective, randomised multicentre study to measure the impact of a learning programme via WM on clinical reasoning in anaesthesiology residents from four university hospitals in France. Residents were randomised into two groups of online teaching: WM-based one and e-learning control group. The WM group benefited from the daily delivery of teaching documents on the instant messenger system and a weekly clinical case supervised by a senior physician. Residents in the control group access to identical records via a traditional e-learning platform. Medical reasoning was self-assessed online by a script concordance test, and medical knowledge was assessed using multiple-choice questions. The residents also completed an online satisfaction questionnaire. The authors found a difference between the WM and control groups for the script concordance test (*p* = 0.006) but no difference for multiple-choice questions. The global satisfaction rate was more significant in the WM than in the control group (*p* = 0.049). Compared to traditional e-learning, the use of WM for teaching residents was associated with worse clinical reasoning despite better global appreciation. The use of WM probably contributes to the dispersion of attention linked to the use of the smartphone. An Indian study evaluated the feasibility and effectiveness of WM in a series of 182 students to provide health education on tobacco and oral cancer as compared to the conventional PowerPoint lessons [32]. Both WM and traditional student groups showed a statistically significant increase in knowledge scores, with substantial improvement in the WM intervention group (*p* = 0.001). However, the validity of WM as a helpful tool in health education strategies has been criticised by other authors [33].

### Clinically related issues

#### Remote monitoring and consultation

Any patient can access healthcare services through WM no matter geographical barriers or local time. WM chatbots are also available for mobile consultation to get customised tips or treatment, especially in the current pandemic. On the other hand, doctors can witness a vast number of customers for their services. The widespread use of fast communication tools reduces the gap between low and high-income countries, allowing cancer patients with limited socioeconomic conditions to receive medical assistance closer to patients living in higher conditions [34]. The communication tools, often WM, are helpful in low- and middle-income countries where the incidence and mortality of cancer are high. Yadav and Yadav [35] reported that WM remote monitoring of surgical wounds in patients operated for thyroid cancer was as effective as face-to-face visits with a higher rate of high satisfaction in patients evaluated via web and avoidance of a median of 930 km travel. The authors stressed how these communication tools might be helpful in low- and middle-income countries where the incidence and mortality of cancer are high. An observational cross-sectional study reported positive data for WM to prevent malignant oral cavity disorders in a rural area of India [36]. Overall, 131 patients were screened for oral potentially malignant diseases using photo capturing five regions of the mouths and send via WM to remote examiners and compared to face-to-face clinical evaluation. The kappa reliability score between the diagnoses, based on photo messaging and clinical oral examination, was 0.68, while sensitivity and specificity were 98% and 52%–64%, respectively.

#### Telepathology

Most of the studies on telepathology have considered results encouraging. However, all concluded that smartphone-assisted telepathology still needs a faster Internet resource and significant improvement in smartphone camera technology to improve the quality and accuracy of the diagnosis. Dixit *et al* [37] reported the use of WM in telepathology in India. A smartphone camera captured 151 cases of fine-needle aspiration and 10 cases of urine cytology from the ocular of a binocular microscope. Investigators sent images to the cytopathologist at a different location. Results of telepathology were compared to direct microscopic evaluation showing an overall intra-observer concordance of 95.6% and 90% for fine-needle aspiration specimens and urine cytology. The authors concluded that further improvement in the smartphone camera resolution and Internet connectivity would enhance the results. Turkish researchers studied 172 specimens to determine intra-observer concordance between traditional microscopic cytopathological diagnoses and diagnoses done on static smartphone images transmitted via WM [38]. This blinded analysis showed an 84% concordance rate, with a 1.000 Kappa agreement in endoscopic ultrasound-guided fine-needle aspiration specimens and only 0.665 in urine cytology (0.665). The authors concluded that WM is a fast way to transmit images but considered mandatory improvements in smartphone camera technology and transfer software. A Brasilian study evaluated the appropriateness and timeliness of telepathology of 23 endoscopic ultrasound-guided fine-needle aspirations specimens evaluated through WM [39]. An initial diagnosis of malignancy was possible in 14 of 23 patients (60.8 %). The current study demonstrated the feasibility of a low-cost, Internet-based telepathology system using WM. WM may be helpful to rapidly achieve a second opinion. A total of 247 oral histopathological specimens received a second opinion by 20 different pathologists using WM [40]. Overall, 98% of 4,795 total second opinions received a concordant diagnosis. The percentage of valid second opinions for malignant tumour specimens was lower than that formulated for cysts of benign or low malignant diseases (75%–85% versus 100%). A positive correlation was observed between correct second opinion and age (*p* = 0.0143) and experience (*p* = 0.0189) of the pathologist. Similar results were reported for gynaecology specimens. Out of 186 gynaecologic pathology cases analysed by conventional microscopy or smartphone camera-derived images and transferred via WM, the smartphone diagnosis was concordant in 179/186 (96.2%) cases [41]. The intra-observer concordance rate was 97.2% and 97.6% for cervical and endometrial myometrial pathology, respectively, and decreased to 80% for ovarian lesions.

#### Online surveys, screening and prevention

WM is a valuable tool to carry out online surveys. An international group of researchers belonging to four scientific societies explored the status of implementing an Enhanced Recovery After Surgery programme in open gynaecologic oncology surgery. The study aimed to provide a worldwide perspective on perioperative practice patterns and decrease the variation in the quality of care [42]. Over 454 respondents representing 62 countries, 37% reported a programme implementation at their hospitals, even if Western industrialised countries were more compliant (33%–38%) than Asian and African ones (10%–19%). Data showed a high adherence rate for venous thrombosis prophylaxis, early postsurgical removal of a urinary catheter and precious mobilisations. Poor adherence to the guidelines included bowel preparation, adoption of modern fasting guidelines, carbohydrate loading, use of nasogastric tubes and peritoneal drains, intra-operative temperature monitoring and early feeding. Longitudinal adherence to cancer screening, critical to the success of any faecal test-based screening programme, is often inadequate. Uy *et al* [43] extensively reviewed 31 out of 2,238 citations concerning cancer screening finding nine papers satisfying inclusion criteria. Five studies examined screening for breast cancer, one for cervical cancer and three for colorectal cancer. Interventions based on sending messages moderately increased adherence to screening programmes which was 0.6%–15.0% higher than for controls. The benefit was observed in various countries, including resource-poor and non-English-speaking populations. Given the lack of data, additional research is needed to quantify this promising intervention’s effectiveness better. Lam *et al* [44] carried out a single-blind, randomised study on reminders for a faecal immunochemical test sent via mobile messengers, such as WM. The study showed a clear improvement of adherence defined as the pick-up and on-time return of tests (80.3% versus 59.3%; *p* < 0.001), and return rate (79.9% versus 57.3%; *p* < 0.001) were significantly higher in the WM group compared with the control group. A study carried out at the Johns Hopkins University aimed to evaluate the barriers in research recruitment among racial/ethnic minorities and immigrants [45]. This community-based study examining correlates of cervical cancer screening behaviours showed that online recruitment via WM was an effective recruitment strategy because it built on existing information-sharing norms within the community. A cross-sectional study carried out in Kuwait reached the same conclusions [46]. WM was used to disseminate a survey on oral cancer risk. Among 404 respondents, the prevalence of oral cancer screening was 7.2, 7.7% among nonsmokers, and 5.4% among smokers. Only 36.6% were aware of oral cancer, with more nonsmokers (38.9%) than smokers (29%). Logistic regression analysis showed that male patients participated more likely to go for screening than females. The likelihood of participation was correlated to age, being individuals in the age group of 25–44 years four times more than other age groups (*p* < 0.012). Walter *et al* [47] studied the effect of a commercially available skin self-monitoring smartphone application among 238 patients with increased risk of melanoma on their decision to seek help for changing skin lesions. Overall, 51 patients (21.4%) had visits regarding skin changes during the 12 months of follow-up, and 157 individuals (66.0%) completed one or more questionnaires for follow-up. There were no significant differences in dermatologic consultation rates, size of superficial spreading lesions or psychological harm evaluated by the Melanoma Worry Scale.

#### Virtual multi-disciplinary tumour board

An informal WM group was created among 25 specialists, including nine urologists, nine oncologists, three urology residents, three radiotherapists, one general practitioner and a group coordinator [48]. This virtual tumour board discussed clinical cases of genitourinary tumours of particular complexity requiring a multi-disciplinary approach. An evaluation questionnaire was sent after 6 months to evaluate the level of appreciation, reporting an average rating score of appreciation was 7.8 (range: 4–10) according to a 10-point Likert scale. The WM consultation was completed in 90% of cases, and in 81.8% of cases, a final agreement on patient management was reached. An average of eight specialists or each case joined the WM-based tumour board, with an average of 17.6 textual interventions for each clinical case. Authors felt WM as a helpful alternative and a rapid complementary communication tool to transfer large amounts of clinical and radiological data.

### Patients’ related issues

#### Patients preference

Cherrez-Ojeda *et al* [49] carried out an anonymous cross-sectional survey study in 500 Ecuadorian cancer patients to assess the use of communication technology and patterns of preferences. Only 43% of participants declared to have web access. WM was the preferred method to receive or ask for information from oncologists in 72% of cases. Age was correlated to the likelihood of using instant messaging systems. Patients aged between 40 and 64 years were more likely to be interested in receiving information through SMS (odds ratio (OR): 5.09, 95% CI: 1.92–13.32), as well as for asking questions to physicians through this same media (OR: 9.78, 95% CI: 3.45–27.67) than the oldest group.

#### Feasibility, useability and acceptability

Australian investigators reviewed the medical literature concerning the feasibility, useability and acceptability of web technology-based interventions [50, 51]. The review also included data concerning smartphone applications among patients’ caregivers. Attrition, recruitment rates and frequency of intervention use were measures of feasibility. Usability was measured by the ease of intervention use and the role of features to minimise errors in use. Acceptability was measured by carers’ perception of the appropriateness of the content and their ability to incorporate the intervention into their daily routines. Six out of the 729 articles examined met the inclusion criteria. Feasibility attrition ranged from 14% to 77%, recruitment rates from 20% to 66% and intervention useability varied across studies. Half of the studies implemented measures to improve useability. Overall, carers rated the content of the interventions as appropriate and reported improved knowledge and communication. Data further demonstrated acceptability as carers preferred the flexibility available with web-based interventions. Although current studies have shown the poor quality of evidence, mobile apps may be an efficient solution for caregivers even if dependent on users’ needs [52]. Patients found this strategy very convenient and cost-effective and suggested to continue this service in the future, even after the lockdown is lifted [70].

#### Cancer awareness and risk reduction

Canadian investigators explored the age differences and degree of confidence with web-based health resources in 371 cancer survivors finding younger age statistically linked to the likelihood of using social media for cancer care [53]. US researchers reviewed 18 studies, including seven randomised trials, using social media among breast cancer women and healthy subjects showing that web-based interventions effectively improve cancer prevention and management [54]. However, the methodology employed to evaluate the impact of social media on cancer-related outcomes needed to be improved in most studies. Authors from the United Arab Emirates reached the same conclusions on the positive role of social media and WM as communication tools to raise awareness and share upcoming breast cancer events and screening [55]. Since many women can discover a breast lump via observation and self palpation, they must be breast cancer aware, i.e., have the knowledge, skills and confidence to detect breast changes and present promptly to a healthcare professional [56]. Pereira *et al* [57] explored the potential of WM as a health education tool to improve women’s knowledge on the risk reduction of breast cancer. The authors examined a total of 293 messages dealing with incidence, risk factors, symptoms, myths, protective factors. Although non-specific for the use of WM, many exciting issues emerged from the analysis of the messages, such as social dynamics, uncertainties, spirituality and web or journal news. Communication tools provide the opportunity to exchange personal experiences with disinhibition. However, the presence of a moderator, either a physician or another figure, is necessary to avoid distractions or disengagement, which represent a possible limitation.

#### Patients’ needs

A descriptive investigation was undertaken at three oncology units to report queries, needs and fears related to the COVID-19 pandemic in patients with cancer and avoid uncontrolled treatment delays or withdrawal, behavioural mistakes and panic [58]. All queries spontaneously delivered through the WM commonly used by patients to communicate with the oncology units were collected and grouped by homology in five categories. Responses to the questions were given according to the Italian Association of Medical Oncology recommendations through WM and subsequent phone calls. Patients were also classified according to the primary tumour site, stage of disease and current treatments. Analysis of the association between these data and queries was carried out. Overall, 446 different patients’ WM conversations were analysed between 1 March and 13 March 2020, and comprised the following: requirement of visit delay by patients undergoing oral therapies or in follow-up, delays in chemotherapy or immunotherapy administration, queries about possible immunosuppression and changes in lifestyle or daily activities. Delay requirements were statistically more frequent among patients with prostate or breast cancer than those with lung or pancreatic cancer. Actions taken by oncologists are also reported. The authors concluded that this experience showed WM to be adequate to give a rapid answer to most queries from patients with cancer in the COVID-19 pandemic scenario. COVID-19 pandemic caused several countermeasures such as strict restriction or complete limitation of hospital visits to relatives. WM was employed to allow family members to participate in clinical rounds in an acute palliative care unit and hospice [59]. Although most of the interviewed family members reported a positive impression, the actual presence bedside was considered irreplaceable. De Camargos *et al* [60] assessed happiness/satisfaction with life and positive and negative effects in 342 cancer patients and 126 informal caregivers compared with healthy people employing WM. In this cross-sectional study, patients and informal caregivers reported at multivariate analysis more positive emotional states than the healthy people despite lower positive affect scores and higher negative affect scores. Although cancer patients experience distressing symptoms and health-related changes in their quality of life, they may report positive emotional states. UK investigators carried out an in-depth qualitative study to explore how women use and experience social media and instant messenger systems, including WM, to self-manage their psychosocial needs and support self-management across the breast cancer continuum [61]. Results showed that such media allowed women living with or after breast cancer to timely self-manage some needs and receive support. However, women experienced social media as empowering and dislocating, as their everyday cancer experiences impacted their engagement. The authors concluded that professionals involved in the cancer health care continuum should improve their awareness of using such tools to initiate value-free discussions and create the space necessary for a tailored and timely self-managed approach to their unique psychosocial needs for women. Recently, the World Health Organization (WHO) created a tool to provide updates and verified data to WhatsApp users, being able to reach more people at once in a simple and effective way. At first glance it looks like a contact like many others saved in the address book but to respond to messages sent by users there is a software ready to provide updates screened directly from the WHO [62].

#### Clinical monitoring

A literature review of 26 articles addressed the use of mobile applications to monitor adherence and support in the self-management of complications associated with chemotherapy treatments [63]. The authors identified 16 different mobile applications, which resulted in an effective monitoring process. These apps should be tested in a real-world setting since the evaluation of efficacy in a scientific programme may represent a possible limitation and does not necessarily mean effectiveness in routine practice. Another systematic review by Brasilian investigators reported the usefulness of mobile apps to spread knowledge for breast cancer patients and symptoms reporting management of treatment-related side effects and self-care promotion [64]. Similar conclusions were reported of breast cancer patients in United Arab Emirates [55]. An Indonesia scoping review analysed the use of mobile apps in the management of home palliative care, reporting they can improve the delivery of care, primarily through the improvement of knowledge among family carers [65]. These statements have been confirmed by a Canadian descriptive, exploratory, proof-of-concept study carried out to evaluate the efficacy of telemedicine in guiding familiar caregivers or nurses in delivering palliate care at distant homes [66]. Patients and caregivers reported a positive effect and convenience of such intervention. The main aspects reported through questionnaires and interviews included communication, logistics, technical issues, trust and insecurity. Among social media, WM was successfully employed in the remote evaluation of haematuria in a series of 212 patients reducing unnecessary costs of services in cases with mild clinical significance [67].

### Pitfalls

Patients with severe or potentially severe clinical picture are not suitable for telemedicine or other communication tool evaluation, including WM, since they most often require a physical examination, protocol-driven procedures and decisive in-hospital interventions. Clinical emergencies, cognitive alterations, comprehension barriers, lack of access to virtual visit technology represent medical situations that necessarily require a face-to-face visit to guarantee adequate assistance. A possible exception is represented by the management at the home of patients in palliative therapy or in a terminal state that can be managed effectively via the web [68, 69]. Australian researchers showed that implementing a palliative care website requires considerable networking skills, resources, commitment and flexibility to adapt to different user groups such as practitioners, other professional figures, allied health professional groups, patients and their caregivers [69].

## Conclusions

Instant messaging systems in cancer care facilitate improved communication and support between patients, caregivers and clinicians, and consequently a better patient care.

These benefits are particularly useful in rural areas and low- and middle-income countries to partially overcome economic and distance barriers to care, although in these countries the availability of the Internet and the costs associated with connectivity and devices may be a limitation.

In addition, in the COVID-19 pandemic scenario, the rapid transition to electronic visits for non-treatment cancer patients provided an immediate benefit by reducing the unnecessary risk of exposure to infection, overcoming the transport barriers faced by patients and caregivers. A deeper understanding of the impact of e-medicine on clinical outcomes, quality of care and access to care is mandatory to delineate the role of telemedicine and communication tools in the post-pandemic era. Clinicians need to carefully manage instant messaging systems to improve patient care and clinical outcomes. However, the unregulated use of WM, the most cited communication tool in healthcare, raises legal, regulatory and ethical concerns. Consequently, there is a huge need for general guidelines, but this is a difficult goal to achieve. In conclusion, although a significant number of articles have studied the role of social networks in connecting patients to health professionals, there is still a lack of scientific information and data on their uses, benefits and limitations in connecting health professionals for professional communication only. The role of instant messaging systems in cancer practice and research needs to be clarified.

## Authors’ contributions

**Conceptualisation:** VG and DP; **methodology:** DP and AF; **formal analysis:** AF, VG and MRV; **writing — original draft preparation:** VG and DP; **writing — review and editing:** all authors have read and agreed to the published version of the manuscript.

## Funding

This research received no external funding.

## Institutional review board statement

Not applicable.

## Conflicts of interest

None of the authors have any personal conflicts of interest to declare.
